# Minimum caseload for cost-effective robotic-assisted surgery: a systematic review

**DOI:** 10.1007/s11701-025-03122-6

**Published:** 2026-02-04

**Authors:** Alice Bartolomeu Garavini, Ana Clara de Carvalho Veludo, Kate Alexander, Juliette Cotte, Scott Leslie, Ruban Thanigasalam, Leani Souza Maximo Pereira, Ana Paula Drummond Lage, Kevin Fritz Arnaiz, Daniel Steffens

**Affiliations:** 1 Surgical Outcomes Research Centre (SOuRCe), Royal Prince Alfred Hospital (RPAH), Sydney, NSW 2050 Australia; 2 Faculty of Medicine and Health, Central Clinical School, The University of Sydney, Sydney, NSW 2050 Australia; 3 Department of Urology, Royal Prince Alfred Hospital, Camperdown, NSW Australia; 4 Department of Urology, Chris O’Brien Lifehouse, Camperdown, NSW Australia; 5 Institute of Academic Surgery, Royal Prince Alfred Hospital, Camperdown, NSW Australia; 6 Department of Urology, Concord Repatriation General Hospital, Concord, NSW Australia; 7 Postgraduate Program in Health Sciences, Faculdade de Ciências Médicas de Minas Gerais (FCMMG), Belo Horizonte, MG Brazil

**Keywords:** Robotic surgery, Health care costs, Da Vinci surgical system, Mako robotic-arm system, Cost-effectiveness

## Abstract

Robotic-assisted surgery (RAS) has been increasingly adopted across surgical specialties due to its potential clinical benefits. However, the high fixed and variable costs associated with robotic platforms raise concerns regarding economic sustainability. Procedural volume is frequently cited as a key determinant of cost-effectiveness; however, reported minimum caseload thresholds vary widely across surgical specialties, robotic platforms, and healthcare settings. This systematic review aims to synthesise the available evidence on cost-effectiveness thresholds and annual surgical volumes associated with the economic viability of the da Vinci and Mako robotic systems. Searches were conducted in MEDLINE (via PubMed), Embase, and the Cochrane Library. Two independent reviewers performed study selection and data extraction. Studies reporting minimum procedural volume thresholds and/or cost-effectiveness outcomes for da Vinci or Mako robotic systems were included. Results were synthesised descriptively and stratified by surgical specialty and robotic platform. Ten studies met the inclusion criteria, encompassing orthopaedic, urologic, colorectal, and head and neck surgery. Reported minimum procedural volume thresholds varied substantially, ranging from approximately 40 to 300 cases per year. This heterogeneity reflected differences in surgical specialty, robotic platform, study design, economic perspective, and underlying modelling assumptions. Across studies, high-volume centres were more consistently associated with favourable cost-effectiveness profiles, whereas findings in low- and intermediate-volume settings were highly variable and context dependent. The cost-effectiveness of robotic-assisted surgery is strongly influenced by procedural volume but remains highly context dependent. No single minimum caseload threshold can be generalised across surgical specialties or robotic platforms. Reported volume thresholds should therefore be interpreted within specific clinical, organisational, and economic contexts when informing decisions regarding investment in robotic surgical systems.

**Supplementary Information:**

The online version contains supplementary material available at 10.1007/s11701-025-03122-6.

## Introduction

The integration of robotic-assisted surgical systems into operating rooms worldwide represents a major innovation in surgical practice [1]. Robotic-assisted surgical platforms such as the Da Vinci Surgical System and the Mako Robotic-Arm Assisted System have increasingly been adopted across multiple specialties due to their potential to enhance precision, reduce invasiveness, and accelerate recovery [1, 2]. Despite these benefits, robotic platforms involve substantial fixed and variable costs, including capital acquisition, maintenance, and consumables [3, 4]. Within the framework of value-based healthcare, where clinical outcomes must be weighed against economic sustainability, these technologies demand rigorous cost-effectiveness evaluation [1].

A key determinant of cost-effectiveness in robotic surgery is procedural volume [1]. Acquisition costs may exceed USD 2 million, with annual maintenance around USD 100,000 and consumables ranging from USD 700–3,200 per case. As such, economic viability is often only achieved when these expenses are distributed across sufficiently high thresholds [3, 4].

Although many studies have examined the cost-effectiveness of individual robotic procedures, there remains limited consolidated evidence on the annual volume required to reach economic break-even points [1]. The definition of minimum procedural thresholds is therefore critical, as it determines whether robotic programs achieve cost-effectiveness or remain financially unsustainable.

Evidence across surgical specialties highlights the complexity of defining a single cost-effectiveness threshold for robotic surgery. Procedural requirements vary substantially according to surgical specialty, case complexity and duration, patient characteristics, and healthcare system structure. As a result, minimum case volume thresholds remain highly heterogeneous across the literature, making it difficult to establish a universal benchmark for economic sustainability.

Despite the above points, defining such thresholds is not merely a methodological issue but a fundamental determinant of whether healthcare systems can justify investing in robotic platforms and sustain their clinical application. To address this gap, we synthesized the available evidence on the cost-effectiveness of robotic-assisted surgery, focusing on the Da Vinci and Mako platforms. These systems dominate clinical practice and the economic literature, providing sufficient data for meaningful comparison of cost-effectiveness thresholds. Other robotic systems were excluded due to limited adoption, heterogeneous applications, and scarce cost-effectiveness evidence, which precluded robust synthesis.

## Methods

### Information sources and search strategy

A comprehensive literature search was performed in PubMed/MEDLINE, Embase, and the Cochrane Central Register of Controlled Trials (CENTRAL) from database inception to May 31, 2025. The search strategy combined controlled vocabulary and free-text terms for robotic-assisted surgery, cost-effectiveness, economic evaluation, minimum threshold, surgical volume, and robotic platforms Da Vinci and Mako. The complete search strategy for each database is provided in Supplementary Appendix 1. In addition, the reference lists of included studies were screened to identify further eligible records.

### Selection process

All retrieved records were exported to Covidence software for deduplication and screening. After removal of duplicates, titles and abstracts were screened independently by two reviewers. Full-text review was performed for all potentially relevant records. Discrepancies were resolved through discussion or, if necessary, consultation with a third reviewer.

### Eligibility criteria and outcomes

Studies were eligible if they evaluated surgical procedures performed with the Da Vinci or Mako robotic platforms across orthopaedic, colorectal, urologic, head and neck, or upper gastrointestinal specialties, compared with conventional approaches (such as open, laparoscopic, or computer-assisted surgery). Studies were only included if they reported on minimum threshold or volume thresholds required for cost-effectiveness refers to the balance between additional costs and health benefits or cost-neutrality which means that robotic surgery incurs equivalent costs compared to conventional approaches. Both randomized controlled trials and prospective or retrospective comparative cohorts were included, as well as decision-analytic economic models (e.g., Markov-based cost-effectiveness analyses). Only articles published in English were considered, while protocols, commentaries, conference abstracts without full data, and studies assessing robotic systems other than Da Vinci or Mako were excluded.

The primary outcome was the minimum caseload threshold required for cost-effectiveness, with justification in terms of economic impact. Eligible economic outcomes included incremental cost-effectiveness ratios (ICERs) expressed as cost per quality-adjusted life year (QALY) gained; cost-utility analyses based on QALYs or life-years gained; cost-minimization analyses, when clinical outcomes were considered equivalent, to identify the least costly option; and cost-difference analyses assessing net cost variations across surgical approaches at the hospital or system level.

### Data collection process and data items

For each included study, two reviewers independently extracted data using a standardized form. The following information was collected: study and population characteristics (author, year, country, sample size, study design, specialty, and procedure type); the robotic platform and comparator technique; and the economic perspective considered (institutional, hospital, payer, or societal). Economic outcomes were also recorded, including incremental cost-effectiveness ratios (ICERs) expressed as cost per quality-adjusted life year (QALYs) gained, reported quality-adjusted life years (QALYs) or life-years gained, direct and indirect cost components, incremental hospital or system costs, and any minimum procedural volume thresholds associated with cost-effectiveness or cost-neutrality. To facilitate comparison, all reported costs were converted to US dollars (USD). Results were synthesized narratively, structured by surgical specialty (colorectal, orthopaedics, urology, head and neck, upper gastrointestinal) and robotic platform (Da Vinci or Mako).

### Risk of bias and quality assessment

Risk of bias was assessed according to study design. The Cochrane Risk of Bias 2 (RoB 2) tool was applied to randomized controlled trials, the Risk Of Bias In Non-randomized Studies of Interventions (ROBINS-I) tool to observational cohort studies, and the Consensus on Health Economic Criteria (CHEC) checklist to decision-analytic models. Assessments were performed independently by two reviewers, with disagreements resolved by consensus. The detailed risk of bias assessment is presented in Supplementary Appendix 2.

### Protocol and registration

This systematic review was prospectively registered on the Open Science Framework (OSF) (10.17605/OSF.IO/J8WKS). The review was conducted in accordance with the PRISMA 2020 statement and the CHEERS reporting guidelines for economic evaluations.

## Results

### Study selection

**Fig. 1:**
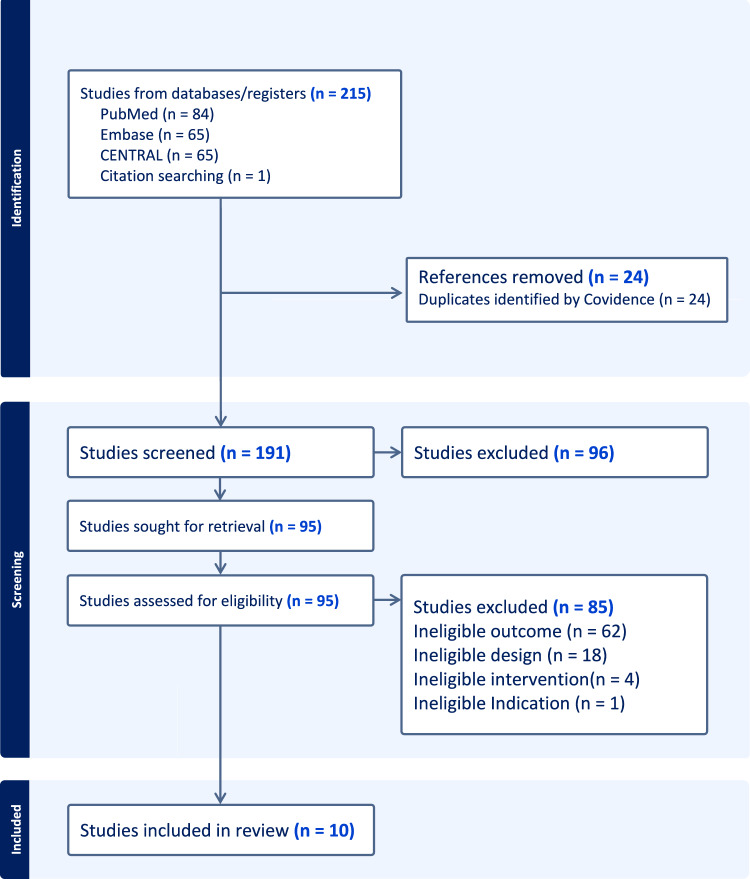
Flow diagram of included studies

A total of 215 records were identified through database searches. After removal of 24 duplicates, 191 records remained for screening. Following title-and-abstract screening, 96 records were excluded. Of the 95 full-text articles assessed for eligibility, 85 were excluded in line with our prespecified ineligibility criteria: outcomes (n = 61), study design (n = 18), intervention (n = 4), and population/indication (n = 2). Ultimately, 10 studies met the inclusion criteria and were included in this systematic review (see Fig. 1).

### Characteristics of the included studies

The 10 included studies, published between 2013 and 2025, were predominantly conducted in high-income countries. The United States contributed the largest number (n = 3, 30%) [5–7], followed by the United Kingdom (n = 3, 30%) [8–10], with additional single studies from France [11], Australia [12], Belgium [13], and Sweden [9] (n = 1 each, 10%). Regarding study design, four studies (40%) employed decision-analytic models, such as Markov analyses and decision trees [5, 7, 13, 14], while six (60%) were comparative cohorts, randomized trials, or retrospective analyses [14]. In terms of surgical specialty, orthopaedics accounted for six studies (60%) [6–8, 10, 12, 13], urology for two (20%) [6, 9], colorectal surgery for one (10%) [11], and head and neck surgery for one (10%) [RUDMIK]. No studies evaluated outcomes across different specialties. The assessed procedures spanned unicompartmental and total knee arthroplasty with the Mako system [5, 7, 8, 10, 12, 13] as well as radical prostatectomy, rectal cancer resection, and transoral robotic surgery (TORS) with the Da Vinci platform [6, 9, 11, 14]. Sample sizes varied substantially from small retrospective cohorts [6, 11, 12] to large national datasets [9] and hypothetical modelled populations [5, 7, 13, 14]. The main features of the included studies, including design, country, surgical specialty, procedure type, and population size, are summarized in Table 1.

**Table 1:** Characteristics of the included studies

Author (year)	Population characteristics	Robotic system	Robotic procedure type (sample size)	Comparator (sample size)	Study design	Diagnosis	Setting (e.g., hospital type)
COLORECTAL							
Fleming (2023)	Country: France Sample size: n = 100 Age: median 64–65 Gender: 29% female	Da Vinci	Robotic TME (R‑TME; n = 50)	TaTME (n = 50)	Prospective comparative cohort	Patients with mid/low rectal cancer undergoing TME	High‑volume tertiary referral center
ORTHOPAEDICS							
TKA							
Hua (2022)	Country: USA Sample size: NR Age: 65 Gender: NR	MAKO	Robot‑assisted total knee arthroplasty (RA‑TKA)	mTKA	Decision‑analytic Markov model	Patients with end‑stage knee osteoarthritis undergoing primary TKA	US hospital perspective (Medicare costs)
Steffens (2022)	Country: Australia Sample size: n = 258 Age: 69 Gender: 60% female	MAKO	Robotic‑assisted TKA (n = 77)	Computer‑navigated TKA (n = 181)	Retrospective cohort, comparative cost analysis	Patients undergoing primary TKA	Tertiary public hospital
Vermue (2021)	Country: Belgium Sample size: NR Age: 67 Gender: NR	MAKO	Robot‑assisted total knee arthroplasty (RA‑TKA)	mTKA	Markov decision analysis	Hypothetical 67‑year‑old patients with end‑stage knee osteoarthritis undergoing TKA	Belgian healthcare system, payer perspective
UKA							
Clement (2023)	Country: UK Sample size: n = 129 Age: 62 Gender: 45% female	MAKO	Robotic‑assisted unicompartmental knee arthroplasty (rUKA; n = 64)	Manual UKA (mUKA; n = 65)	Prospective, double‑blind RCT	Patients with medial compartment knee osteoarthritis eligible for UKA	Tertiary referral center
Moschetti (2016)	Country: USA Sample size: NR Age: 65 Gender: NR	MAKO	Robotic‑assisted unicompartmental knee arthroplasty	Manual unicompartmental knee arthroplasty (UKA)	Decision‑analytic Markov model	Patients with unicompartmental OA eligible for UKA	US healthcare system
Blyth (2025)	Country: UK Sample size: n = 129 Age: NR Gender: NR	MAKO	Robotic UKA (rUKA; n = 64)	Manual UKA (mUKA; n = 65)	RCT	Patients with medial unicompartmental osteoarthritis of the knee undergoing UKA	UK NHS, multicenter
UROLOGY							
Hyams (2013)	Country: USA Sample size: n = 4064 Age: 59 Gender: 0% female	Da Vinci	Robot‑assisted laparoscopic radical prostatectomy (n = 1,499)	Open radical retropubic prostatectomy (RRP; n = 2,565)	Retrospective analysis	Men undergoing radical prostatectomy (RALP or RRP)	Acute‑care hospital
Forsmark (2018)	Country: Sweden Sample size: n = 2638 Age: 60 Gender: 0% female	Da Vinci	Robot‑assisted laparoscopic radical prostatectomy (RALP; n = 1,835)	Open retropubic radical prostatectomy (RRP; n = 803)	Prospective, controlled	Men undergoing radical prostatectomy for prostate cancer	14 Swedish hospitals (public and for‑profit), national trial
HEAD & NECK							
Rudmik (2015)	Country: Mixed cohort Sample size: NR Age: NR Gender: NR	Da Vinci	Transoral Robotic Surgery	Intensity‑Modulated Radiotherapy (IMRT)	Decision tree + Markov model	Patients with early stage OPSCC (T1–T2 N0 M0)	USA, third‑party payer (insurance‑based)

TME = Total Mesorectal Excision; R‑TME = robotic TME; TaTME = Transanal TME; UKA = Unicompartmental Knee Arthroplasty; rUKA = robotic UKA; mUKA = manual UKA; RCT = Randomized Controlled Trial; TKA = Total Knee Arthroplasty; RA‑TKA = robotic‑assisted TKA; mTKA = manual TKA; NR = Not Reported; NHS = National Health Service; RALP = Robot‑assisted Laparoscopic Radical Prostatectomy; RRP = Open Retropubic Radical Prostatectomy; IMRT = Intensity‑Modulated Radiotherapy; OPSCC = Oropharyngeal Squamous Cell Carcinoma

### Outcomes

All 10 included studies conducted economic evaluations comparing robotic-assisted procedures with conventional techniques.

**Minimum procedural volume threshold**. The minimum thresholds identified across studies are summarized in Table 2.

**Table 2:** Minimum annual procedural volume thresholds reported for cost-effectiveness of robotic assisted surgery

Author (year)	Minimum threshold reported	Threshold justification
COLORECTAL		
Fleming (2023)	> 200 rectal cancer surgeries/year	High-volume centre; Capital/maintenance amortised over 400 cases
ORTHOPAEDICS		
Clement (2023)	≥ 300 cases/year for cost-effectiveness; > 900/year for cost-neutrality (when excluding septic revision)	Cost per QALY fell below $27,114 when ≥ 300 cases/year were performed; rUKA became cost-neutral at > 900/year when excluding septic revision; influenced strongly by consumable costs
Hua (2022)	≥ 49 cases/year	> 49 case/ year.; economies of scale offset robot costs
Moschetti (2016)	≥ 94 cases/year required for cost-effectiveness at $50,000/QALY (WTP threshold)	Sensitivity analysis showed strong dependence on annual hospital volume and robotic system costs; only high-volume centers achieved cost-effectiveness
Steffens (2022)	238 RA-TKAs/year required for cost equivalence	Cost equivalence projected after 23 months of implementation or when performing 238 robotic-assisted TKAs annually; reflects amortisation of capital costs and increased utilisation of the robotic system
Vermue (2021)	≥ 253 RA-TKAs/year per robot	Cost-effectiveness only if ≥ 253/year, based on sensitivity analyses of revision rates + costs
Blyth (2025)	≥ 300 robotic UKAs/year (cost-saving when including all reinfection costs) > 800 cases/year (cost-saving when excluding reinfection costs)	Higher case volumes reduce fixed cost per patient; reinfection risk sensitivity significantly affects ICER
UROLOGY		
Hyams (2013)	High-volume surgeon = > 40 cases/year/ surgeon High-volume hospital = > 60 cases/year/ hospital	High surgeon volume reduced costs for both RRP and RALRP; high hospital volume reduced costs for RALRP only. Even at highest volume hospitals, RALRP remained more expensive than RRP
Forsmark (2018)	50–500 cases/ year (cost-saving > 400 cases in non-for-profit hospital)	Based on volume-outcome relationship and sensitivity analyses
HEAD AND NECK		
Rudmik (2015)	≥ 150–200 robotic cases/year	150–200 cases/yeas threshold extrapolated from robotic prostatectomy literature due to lack of TORS- specific data

QALY = Quality-Adjusted Life Year; WTP = Willingness-To-Pay; RA-TKA = robotic-assisted Total Knee Arthroplasty; TKA = Total Knee Arthroplasty; UKA = Unicompartmental Knee Arthroplasty; rUKA = robotic UKA; mUKA = manual UKA; ICER = Incremental Cost-Effectiveness Ratio; RALRP = Robot-Assisted Laparoscopic Radical Prostatectomy; RRP = Open Retropubic Radical Prostatectomy; TORS = Transoral Robotic Surgery

#### Da Vinci robotic system

##### Colorectal surgery

In the single study reporting on patients with mid/low rectal cancer undergoing robotic TME was reported to be cost-effective in high-volume centres performing more than 200 resections per year, reflecting the need for sufficient procedural throughput to offset capital and maintenance costs [11].

##### Urology

**Fig. 2:**
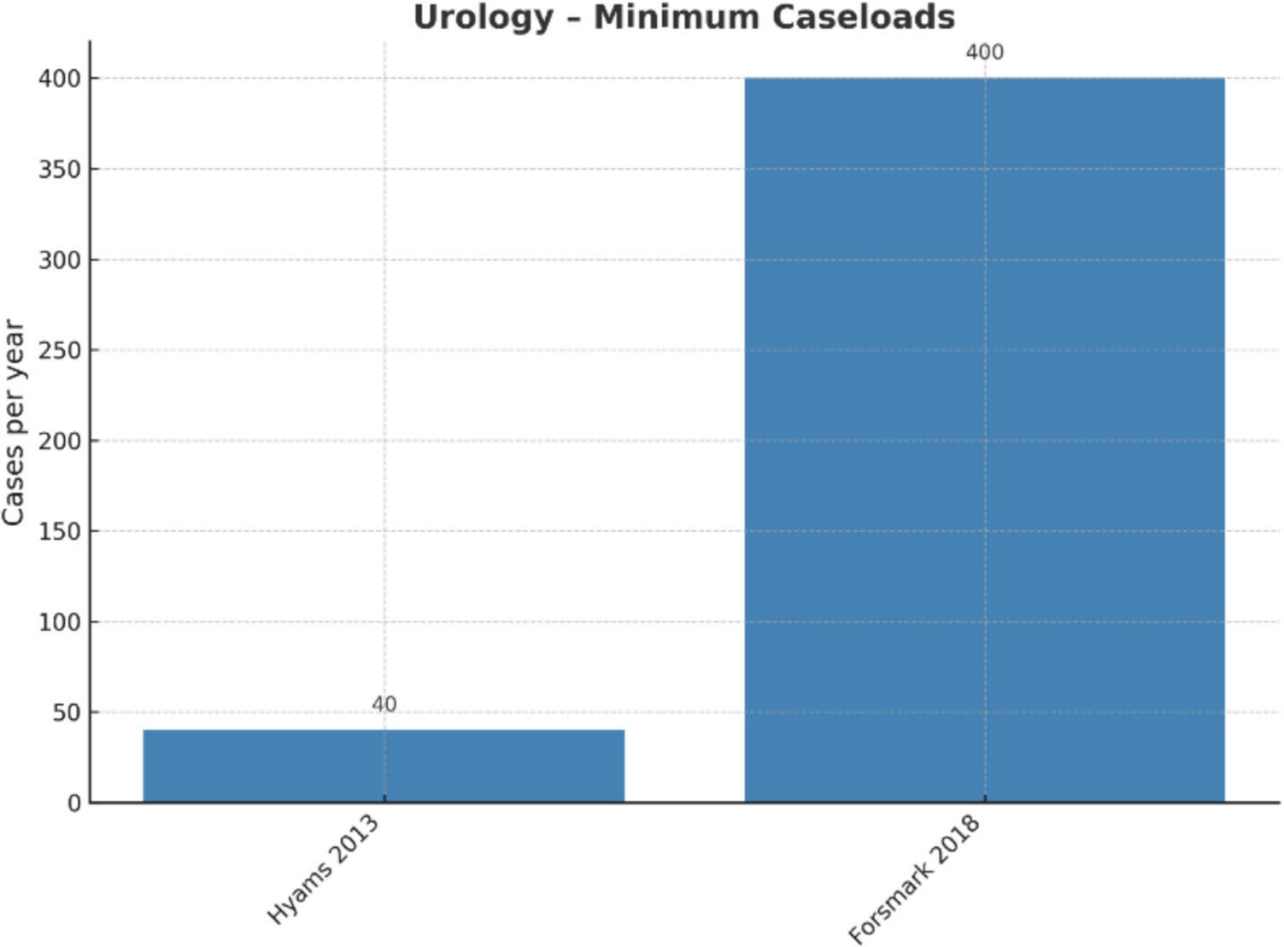
Urology: minimum caseloads

Robotic prostatectomy remained more costly than open surgery, although higher surgeon (> 40 cases/year) and hospital volumes (> 60 cases/year) reduced incremental costs [6, 9] (see Fig. 2).

##### Head and neck surgery

Transoral robotic surgery was not cost-effective compared with Intensity-Modulated Radiation Therapy (IMRT), except potentially in centralised centres (specialised tertiary referral hospitals concentrating complex procedures) performing 150–200 robotic cases annually, where fixed costs may be diluted across a larger caseload [14].

#### Mako robotic system

##### Total knee arthroplasty

**Fig. 3:**
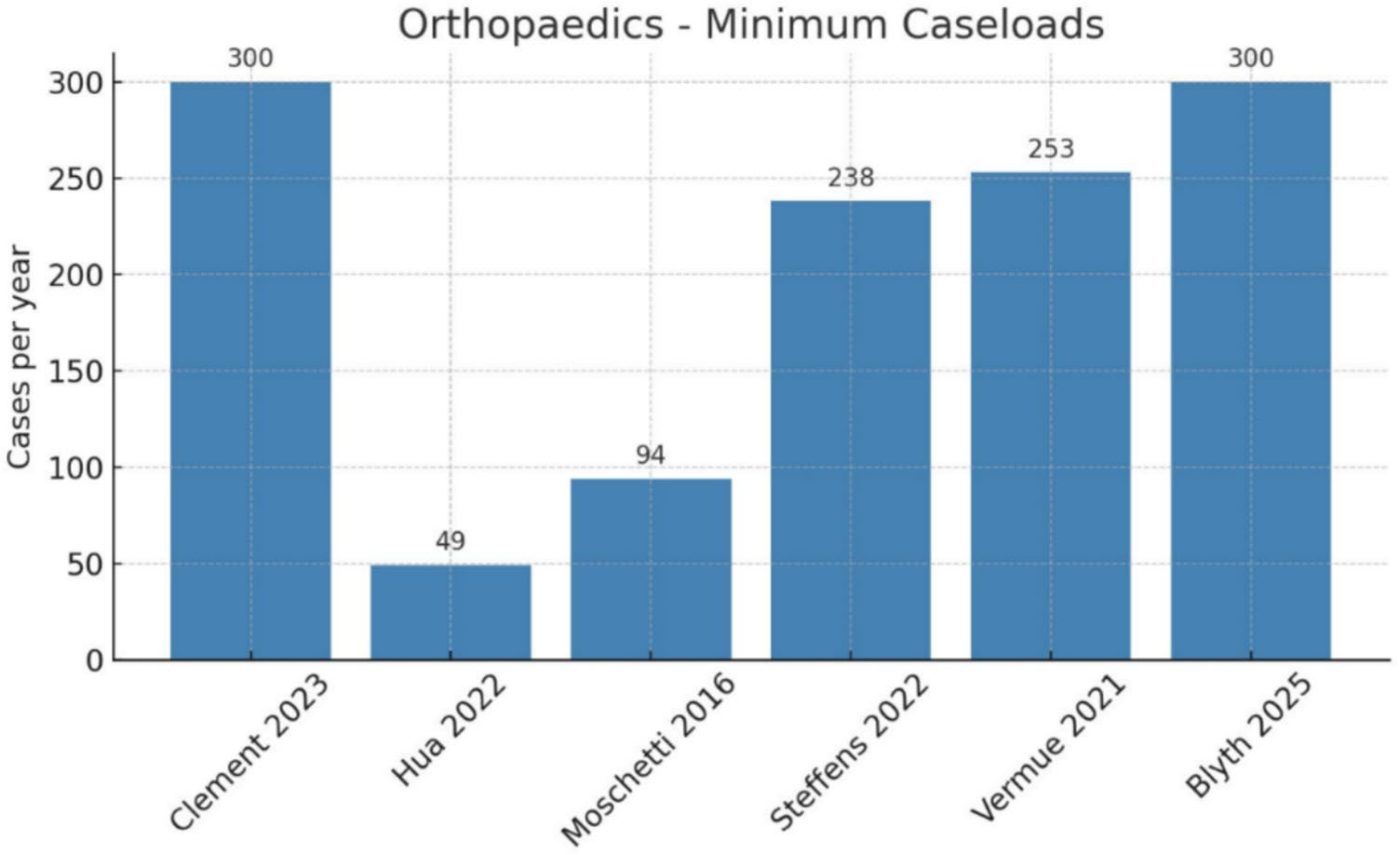
Orthopaedics: minimum caseloads

Six studies evaluated robotic knee arthroplasty, reported separately for total and unicompartmental procedures [5, 7, 8, 10, 12, 13, 15] (see Fig. 3).

- Total knee arthroplasty (TKA): Thresholds for cost-effectiveness ranged from approximately 49 procedures per year [5] to 300 or more annually [10]. Some analyses indicated cost-neutrality only at volumes greater than 900 cases per year [8].
- Unicompartmental knee arthroplasty (UKA): Cost-effectiveness results were more variable, with lower annual thresholds reported in certain models, but overall trends similarly favored by high-volume centers.

Across both procedures, results consistently showed that only high-volume centers achieved cost-effectiveness, whereas low-volume institutions incurred significant incremental costs.

##### Cost effectiveness

Most reported incremental cost-effectiveness ratios (ICERs) and/or cost differences per QALY. Reported cost-effectiveness outcomes and minimum procedural volume thresholds varied considerably across specialties and study designs. These findings are synthesized in Table 3.

**Fig. 4:**
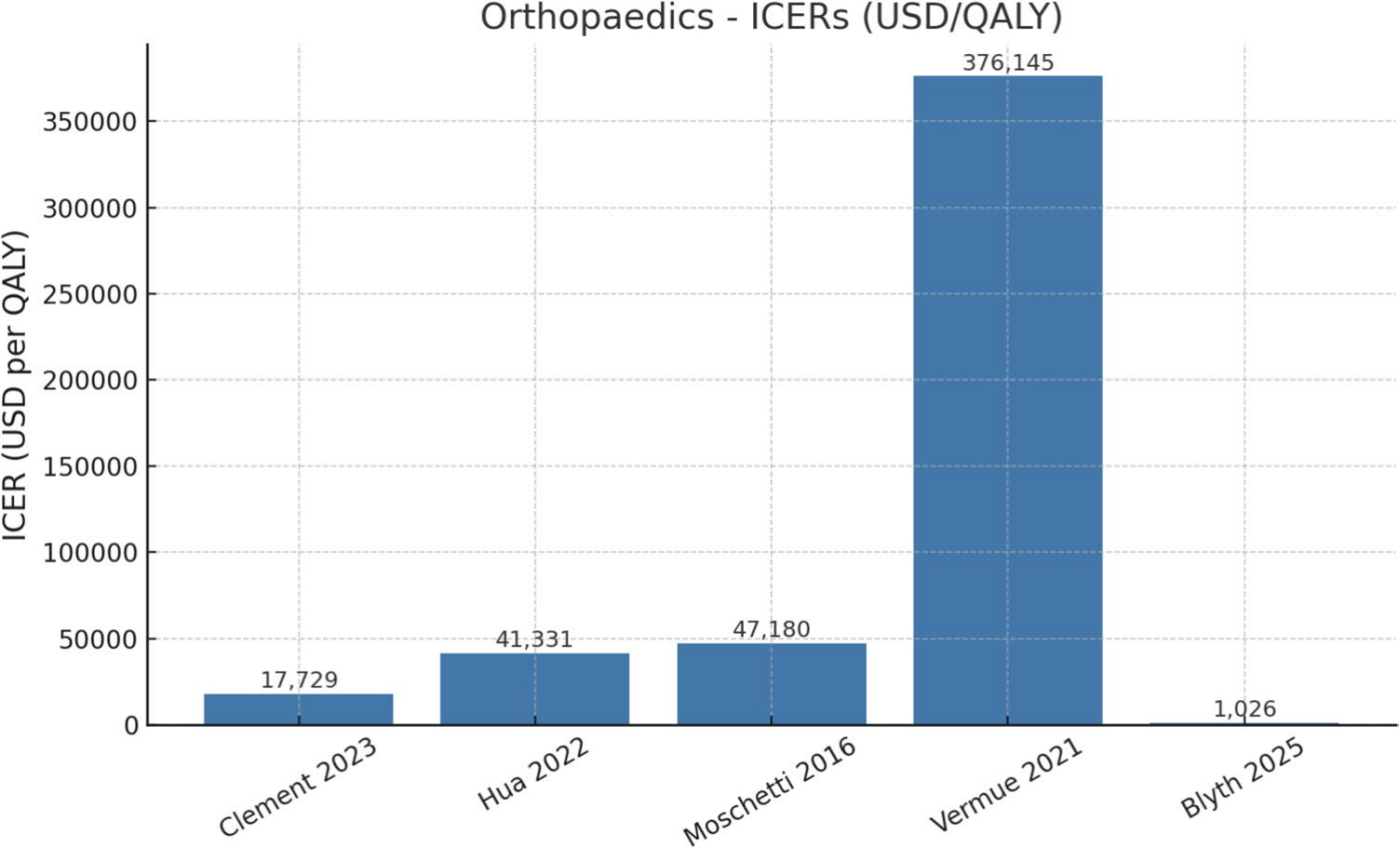
Orthopaedics: ICERS (USD/QALY)

- Cost-effective: Several studies in orthopaedics [5, 7, 8, 10, 13] reported that robotic knee arthroplasty could be cost-effective under defined volume thresholds, often below the widely cited willingness-to-pay (WTP) standard of approximately USD 30,000–45,000 per QALY (see Fig. 4).
- Not cost-effective: Studies in urology [6, 9] and head and neck surgery [14] generally concluded that robotic approaches were more expensive than comparators, with ICERs well above accepted thresholds.
- Conditional cost-effectiveness: Some analyses [5, 7, 12, 13] suggested that robotic procedures could approach cost neutrality or become cost-effective only if annual case volumes exceeded critical thresholds, ranging from > 49 to > 300 procedures per year.

**Table 3:** Reported cost-effectiveness thresholds and economic outcomes

Author(s) (year)	ICER (USD/QALY)	Cost difference (robotic vs. comparator)	QALYs gained	Cost-effectiveness threshold used	Conclusion (cost-effective)
COLORECTAL					
Fleming (2023)	NR	R-TME—TaTME = $153	NR	NR	Both R-TME and TaTME considered cost-effective in high-volume practice
ORTHOPAEDICS					
Clement (2023)	$17,729/ QALY (5 years, 400 cases/ year if septic revision included) $70,706/ QALY if excluded septic revision	RA-UKA—Manual UKA = $212	0.012	$27,114/QALY (NICE; $40,671 explored)	Cost-effective, if septic revision included (ICER $17,729/QALY) Not cost-effective, if septic revision excluded (ICER > $51,516/QALY even at high volume)
Hua (2022)	$41,331/QALY	RA-TKA—mTKA = $747	0.01	$50,000/QALY	Cost-effective at hospitals performing ≥ 49 RATKAs/year; not cost-effective in lower-volume hospitals; overall ~ 50% probability of being cost-effective at WTP $50,000/QALY in sensitivity analysis
Moschetti (2016)	$47,180/QALY—at 100 cases/year	RA-UKA—Manual UKA = $2,743	0.06	$50,000/QALY	RA-UKA cost-effective only at high-volume centers (≥ 94/year), not cost-effective in low- or medium-volume hospitals
Steffens (2022)	NR	RA-TKA—CN-TKA = $1,848	NR	NR	RA-TKA had comparable in-hospital coasts to computer-navigated TKA, but became significantly more expensive when capital/maitenance costs were included. Potential cost equivalence projected with higher procedural volumes or long-term use
Vermue (2021)	~ 376,145/QALY at 70 cases/year	RA-TKA more costly than manual TKA	Minimal incremental QALYs	$50,000/QALY (Belgian payer perspective)	Not cost-effective at standard volumes; only becomes cost-effective if ≥ 253 RA-TKAs/year per robot
Blyth (2025)	$1,026/QALY	rUKA—mTKA ~ $652	0.186	$27,114/QALY	rUKA had lower reintervention and revision risk, higher QALY gain, and was the dominant/cost-saving strategy at ≥ 300 cases/year; ICER well below NICE threshold
UROLOGY					
Hyams (2013)	NR	RALRP—RRP = $ 3,900	NR	NR	No — RALRP remained more costly than RRP even at high-volume hospitals; however, high surgeon/hospital volume significantly reduced cost
Forsmark (2018)	NR	RALP—RRP = $ 3,837	NR	Annual thresholds (50–500/year)	Not cost-effective under base case (RALP was more expensive than RRP)
HEAD AND NECK					
Rudmik (2015)	$165,300/QALY	TORS—IMRT = $ 4,959	0.03	$50,000–150,000/QALY	Not cost-effective; IMRT favored; TORS only potentially valuable in high-volume, centralized centers

ICER = Incremental Cost-Effectiveness Ratio; USD/QALY = United States Dollars per Quality-Adjusted Life Year; QALY = Quality-Adjusted Life Year; R-TME = Robotic Total Mesorectal Excision; TaTME = Transanal Total Mesorectal Excision; RA-UKA = robotic-assisted Unicompartmental Knee Arthroplasty; mUKA = manual UKA; RA-TKA = robotic-assisted Total Knee Arthroplasty; mTKA = manual TKA; CN-TKA = computer-navigated TKA; WTP = Willingness-To-Pay; NICE = National Institute for Health and Care Excellence; RALRP = robot-assisted laparoscopic radical prostatectomy; RRP = open retropubic radical prostatectomy; TORS = Transoral Robotic Surgery; IMRT = Intensity-Modulated Radiotherapy

### Risk of bias

Overall, most decision-analytic models demonstrated a low risk of bias, particularly in reporting transparency and consistency of model assumptions. In contrast, real-world cohort studies frequently showed a higher risk of bias, mainly due to incomplete adjustment for confounders and limited follow-up. Across study designs, common strengths included clear description of populations and interventions, whereas weaknesses were often observed in sensitivity analyses and cost reporting. A detailed appraisal of risk of bias is presented in Supplementary Appendix 2.

### Results of individual studies

Time horizons varied, with decision-analytic models often adopting 5–10-year projections, while real-world cohorts provided shorter-term analyses. Perspectives included hospital, payer, and healthcare system viewpoints, leading to heterogeneity in results.

## Discussion

This systematic review demonstrates that the wide variation in reported minimum caseload thresholds for cost-effective robotic-assisted surgery is itself a central finding rather than a limitation of the evidence. Across surgical specialties and robotic platforms, reported thresholds differ by an order of magnitude. While procedural volume consistently emerged as an important determinant of economic viability, no single, universally applicable minimum caseload threshold can be defined. Instead, reported volume estimates should be interpreted within specific clinical, organisational, and economic contexts.

Across specialties, surgical volume consistently emerged as an important determinant of economic viability. Robotic procedures were more likely to be cost-effective in high-throughput centres, where the fixed costs of acquisition and maintenance could be diluted across a greater number of cases. In colorectal surgery, for example, robotic total mesorectal excision was considered cost-effective only when performed in centralised, high-volume centres, underscoring the importance of institutional thresholds in offsetting fixed costs. In orthopaedics, model assumptions yielded substantial heterogeneity in projected thresholds, partly reflecting differences in modelling strategies and the inclusion of two distinct procedures (total and unicompartmental knee arthroplasty). Nevertheless, all studies converged on the need for substantial procedural volumes, reinforcing the sensitivity of cost-effectiveness estimates to modelling choices and comparators.

Importantly, these findings should not be interpreted as directly comparable across robotic platforms or surgical specialties. The predominance of orthopaedic studies based on the Mako system limits comparability with da Vinci–based abdominal and pelvic procedures, and even within da Vinci–assisted surgery, minimum cost-effective volume thresholds differ between urological, colorectal, and head and neck procedures. Pooling results across platforms or specialties may therefore obscure clinically meaningful differences and contribute to apparent uncertainty in reported thresholds.

In contrast, in urology and head and neck surgery, robotic approaches generally remained more costly than conventional alternatives, even in hospitals or surgeons with higher caseloads, suggesting that improvements in precision and clinical outcomes have not yet translated into consistent economic benefits. Urologic studies indicated that higher surgeon (>40 cases/year) and hospital volumes (>60 cases/year) reduced incremental costs, whereas head and neck surgery demonstrated potential cost-effectiveness only in highly centralised centres performing approximately 150–200 robotic cases annually. These patterns further highlight that minimum volume thresholds are not uniform across surgical fields.

Collectively, these findings have important implications for service planning and health policy. Investment in robotic platforms appears most justified in high-volume centres, where procedural thresholds can offset high fixed and maintenance costs, translating into improved cost-effectiveness and potentially better outcomes. Furthermore, strategies such as centralisation of services and platform sharing may help extend the economic benefits of robotic surgery beyond selected institutions.

Quality-adjusted life years (QALYs) are commonly used in health economic evaluations to integrate both survival and health-related quality of life into a single outcome measure, allowing comparison across different interventions and clinical settings. Their use facilitates decision-making by relating incremental costs to incremental health gains through cost per QALY estimates. However, in surgical contexts, QALYs may incompletely capture short-term outcomes that are highly relevant to clinicians and hospital administrators, such as operative time, length of stay, perioperative complications, and learning-curve effects, which may partly explain variability in reported cost-effectiveness estimates.

Importantly, the primary contribution of this systematic review is not the identification of a single minimum caseload threshold for robotic-assisted surgery, but rather the demonstration of why such a universal threshold is unlikely to be appropriate. By synthesising evidence across multiple surgical specialties and robotic platforms, this review highlights the extent of methodological, clinical, and organisational heterogeneity underlying reported cost-effectiveness estimates. These findings underscore that economic evaluations of robotic surgery must be interpreted within platform- and specialty-specific contexts and reinforce the need for more standardised, transparent, and context-sensitive approaches to economic assessment in future research. By clarifying the strengths and boundaries of existing evidence, this review provides a framework to guide more informed decision-making by policymakers, hospital administrators, and clinicians considering investment in robotic surgical systems.

### Study limitations

This systematic review has several limitations that should be acknowledged. First, the included studies demonstrated substantial methodological heterogeneity, with variability in study design, economic perspective, outcome measures (including QALYs, direct hospital costs, and cost differences), and modelling assumptions, which precluded quantitative synthesis or meta-analysis. Second, most studies were conducted in high-income healthcare systems, limiting the generalisability of findings to low- and middle-income settings and to health systems with different reimbursement structures and resource constraints. Third, many evaluations relied on decision-analytic models rather than prospective, multicentre real-world data, which may reduce external validity and limit applicability to routine clinical practice. In addition, cost-effectiveness was commonly assessed at the procedure level, whereas in practice the economic sustainability of robotic platforms depends on aggregate caseload across multiple specialties within an institution. Finally, the review did not directly assess clinical outcomes, surgeon learning curves, or institutional decision-making processes, which may further influence both costs and value in real-world implementation of robotic surgery.

## Conclusion

This systematic review demonstrates that the cost-effectiveness of robotic-assisted surgery using the Da Vinci and Mako platforms is influenced by procedural volume, with reported thresholds varying substantially across specialties. These findings highlight the importance of aligning robotic surgery implementation with institutional capacity, ensuring that innovation translates into both clinical and economic value.

## Supplementary Information

Below is the link to the electronic supplementary material.

Supplementary file1

## Data Availability

No datasets were generated or analysed during the current study.
