# Supplementary material for: Minimum caseload for cost-effective robotic-assisted surgery: a systematic review
Source: J Robot Surg. 2026 Feb 4;20(1):216. doi: 10.1007/s11701-025-03122-6 (PMC12868110; doi:10.1007/s11701-025-03122-6)
Supplement: Supplementary file 1 — Supplementary file1 [file 11701_2025_3122_MOESM1_ESM.docx]

Supplementary Appendix 1. Search Strategy

1. PubMed(MEDLINE)
   ("Robotic Surgical Procedures"[MeSH] OR "robotic surgery" OR "robot-assisted surgery" OR "da Vinci" OR "Mako") AND ("Cost-Benefit Analysis"[MeSH] OR "cost-effectiveness" OR "economic evaluation" OR "healthcare cost" OR "value-based healthcare") AND ("Caseload"[MeSH] OR "minimum caseload" OR "surgical volume" OR "volume threshold" OR "learning curve")
   Filters applied: None (no language or date restrictions).
2. Embase
   ('robot assisted surgery'/exp OR 'robotic surgery' OR 'da Vinci' OR 'Mako')
   AND ('cost effectiveness analysis'/exp OR 'cost-effectiveness' OR 'economic evaluation' OR 'value-based healthcare' OR 'healthcare cost') AND ('surgical volume'/exp OR 'minimum caseload' OR 'volume threshold' OR 'learning curve')
   Limits: None (no language or date restrictions).
3. Cochrane Library
   ("robotic surgery" OR "robot-assisted surgery" OR "da Vinci" OR "Mako")
   AND ("cost-effectiveness" OR "economic evaluation" OR "healthcare cost" OR "value-based healthcare") AND ("minimum caseload" OR "surgical volume" OR "volume threshold" OR "learning curve")
   No language or period filters applied.

**Supplementary Appendix 2.** **Risk of bias assessment of included studies**

ROBINS-I – Risk of Bias Assessment (Cochrane 2016) – Multi‑study Grid

| Signaling Questions (ROBINS‑I 2016) | Steffens  (2022) | Fleming (2023) | Forsmak (2018) | Hua (2022) | Moschetti (2016) | Rudmik (2015) | Vermue (2021) |
| --- | --- | --- | --- | --- | --- | --- | --- |
| D1. Bias due to confounding |  |  |  |  |  |  |  |
| 1.1 Were prognostic/confounding factors balanced or appropriately adjusted for? | ✖️ | ✖️ | ✔️ | ✖️ | ✖️ | ✖️ | ✖️ |
| 1.2 Were all important confounders measured validly and reliably? | ✔️ | ✔️ | ✔️ | ? | ✔️ | ✔️ | ✔️ |
| 1.3 Were appropriate methods used to control for confounding (e.g., matching, weighting, regression)? | ✖️ | ✖️ | ✔️ | ✔️ | ✔️ | ✖️ | ✔️ |
| 1.4 Is there likely residual confounding (e.g., unmeasured/poorly measured confounders)? | ✔️ | ✔️ | ✔️ | ✔️ | ✔️ | ✔️ | ✔️ |
| 1.5 Did time-varying confounding receive appropriate adjustment (if applicable)? | ✖️ | ✖️ | ✖️ | ✖️ | ✖️ | ✖️ | ✖️ |
| D1. Bias due to confounding – Domain-level judgment | Serious | Serious | Moderate | Serious | Serious | Serious | Serious |
| D2. Bias in selection of participants into the study |  |  |  |  |  |  |  |
| 2.1 Were participants selected into the study in a way that avoided baseline imbalances related to intervention and outcome? | ✖️ | ✖️ | ✔️ | ? | ? | ✔️ | ? |
| 2.2 Were inclusion/exclusion criteria applied equally to all groups? | ✔️ | ✔️ | ✔️ | ? | ? | ✔️ | ? |
| 2.3 Could selection of participants have been related to both intervention and outcome? | ✔️ | ✔️ | ✖️ | ? | ? | ✖️ | ? |
| D2. Bias in selection of participants into the study – Domain-level judgment | Serious | Serious | Moderate | Not applicable | Not applicable | Low | Not applicable |
| D3. Bias in classification of interventions |  |  |  |  |  |  |  |
| 3.1 Was intervention status classified correctly for all participants? | ✔️ | ✔️ | ✔️ | ✔️ | ✔️ | ✔️ | ✔️ |
| 3.2 Was misclassification of intervention status unrelated to outcome (non‑differential)? | ✔️ | ✔️ | ✔️ | ✔️ | ✔️ | ✔️ | ✔️ |
| 3.3 Was knowledge of the intervention well documented at the time of classification? | ✔️ | ✔️ | ✔️ | ✔️ | ✔️ | ✔️ | ✔️ |
| D3. Bias in classification of interventions – Domain-level judgment | Low | Low | Low | Low | Low | Low | Low |
| D4. Bias due to deviations from intended interventions |  |  |  |  |  |  |  |
| 4.1 Were there deviations from the intended intervention related to trial context or patient preference? | ✖️ | ✖️ | ✖️ | ? | ? | ✖️ | ? |
| 4.2 Were co-interventions, adherence, and exposure similar between groups or appropriately adjusted for? | ✔️ | ✔️ | ✔️ | ? | ? | ? | ? |
| 4.3 Were appropriate analytic methods used to estimate the effect of assignment/exposure (e.g., ITT, per‑protocol with adjustment)? | ✖️ | ✖️ | ✔️ | ✔️ | ✔️ | ✖️ | ✔️ |
| D4. Bias due to deviations from intended interventions – Domain-level judgment | Moderate | Moderate | Low | Low | Low | Moderate | Low |
| D5. Bias due to missing data |  |  |  |  |  |  |  |
| 5.1 Were outcome data available for all, or nearly all, participants? | ✔️ | ✔️ | ✔️ | ✔️ | ✔️ | ✔️ | ✔️ |
| 5.2 Were reasons for missing data balanced and unrelated to true outcome? | ✔️ | ✔️ | ✔️ | ? | ? | ✔️ | ? |
| 5.3 Were appropriate methods used to handle missing data (e.g., multiple imputation, weighting)? | ? | ✔️ | ✔️ | ? | ✔️ | ✔️ | ✔️ |
| D5. Bias due to missing data – Domain-level judgment | Low | Low | Low | Low | Low | Low | Low |
| D6. Bias in measurement of outcomes |  |  |  |  |  |  |  |
| 6.1 Were outcome measures appropriate, valid, and applied equally across groups? | ✔️ | ✔️ | ✔️ | ✔️ | ✔️ | ✖️ | ✔️ |
| 6.2 Were outcome assessors blinded to intervention status, or unlikely to be influenced by knowledge of it? | ? | ✖️ | ✔️ | ? | ? | ✔️ | ? |
| 6.3 Could measurement or ascertainment of outcomes differ between groups? | ✖️ | ✖️ | ✖️ | ✖️ | ✖️ | ? | ✖️ |
| D6. Bias in measurement of outcomes – Domain-level judgment | Low | Low | Low | Low | Low | Serious | Serious |
| D7. Bias in selection of the reported result |  |  |  |  |  |  |  |
| 7.1 Were analyses specified in advance or supported by a protocol/analysis plan? | ? | ? | ✔️ | ✖️ | ✖️ | ? | ✖️ |
| 7.2 Were multiple eligible outcomes/measurements/analyses avoided or transparently reported? | ✔️ | ✔️ | ✔️ | ? | ? | ✔️ | ✔️ |
| 7.3 Is there evidence of selective reporting of results? | ✖️ | ✖️ | ✖️ | ✔️ | ✔️ | ✔️ | ✔️ |
| D7. Bias in selection of the reported result – Domain-level judgment | Moderate | Moderate | Low | Moderate | Moderate | Serious | Serious |
| OVERALL RISK OF BIAS  (Low / Moderate / Serious / Critical / No information) | Serious risk of bias | Serious risk of bias | Moderate risk of bias | Serious risk of bias | Serious risk of bias | Serious risk of bias | Serious risk of bias |

# RoB 2 – Risk of Bias Assessment Template (Cochrane 2019)

| **Signaling Questions** | **Clement**  **(2023)** | **Hyams**  **(2013)** | **Blyth**  **(2025)** |
| --- | --- | --- | --- |
| 1.1 Was the allocation sequence random? | ✔️ | ✖️ | ✔️ |
| 1.2 Was the allocation sequence concealed until participants were enrolled? | ✔️ | ✖️ | ? |
| 1.3 Did baseline differences suggest a problem with randomization? | ✔️ | ✖️ | ✔️ |
| 2.1 Were participants aware of their assigned intervention? | ✔️ | ✔️ | ✖️ |
| 2.2 Were carers and trial staff aware of participants’ intervention? | ✖️ | ✔️ | ✖️ |
| 2.3 Were there deviations from intended intervention due to the trial context? | ✔️ | ✖️ | ✔️ |
| 2.4 Was an appropriate analysis used to estimate the effect of assignment? | ✔️ | ✔️ | ? |
| 2.5 Was this analysis likely to be substantially biased? | ✔️ | ✔️ | ✔️ |
| 3.1 Were data for this outcome available for all, or nearly all, participants? | ✔️ | ✔️ | ? |
| 3.2 Is there evidence that the result was not biased by missing outcome data? | ✔️ | ✔️ | ? |
| 3.3 Could missingness in the outcome depend on its true value? | ✖️ | ✔️ | ? |
| 3.4 Is it likely that missingness in the outcome depended on its true value? | ✔️ | ✔️ | ? |
| 4.1 Was the method of measuring the outcome inappropriate? | ✔️ | ✔️ | ✔️ |
| 4.2 Could measurement have differed between intervention groups? | ✔️ | ✖️ | ✔️ |
| 4.3 Were outcome assessors aware of the intervention received? | ✔️ | ✔️ | ✖️ |
| 4.4 Could assessment of the outcome have been influenced by knowledge of the intervention? | ✔️ | ✔️ | ✔️ |
| 4.5 Is it likely that assessment was influenced by knowledge of the intervention? | ✔️ | ✔️ | ✔️ |
| 5.1 Were the data analysed in accordance with a prespecified analysis plan? | ✔️ | ✖️ | ? |
| 5.2 Were there multiple eligible outcome measurements (e.g., scales, definitions)? | ✔️ | ✔️ | ✔️ |
| 5.3 Were there multiple eligible analyses of the data? | ✔️ | ✖️ | ✔️ |
| Overall risk of bias judgment | Low risk of bias | Some concerns | Some concerns |

Additional notes: The search was adapted to the syntax of each database; Equivalent strategies were used to identify additional studies through citation tracking and reference lists; No publication filters were applied to maximize sensitivity.
